# Incidence of Dengue fever, serotypes, clinical features, and laboratory markers: a case study of 2019 outbreak at district Shangla, KP, Pakistan

**DOI:** 10.4314/ahs.v22i1.61

**Published:** 2022-03

**Authors:** Abid Ur Rehman, Faheem Anwar, Muhammad Tayyab, Ihteshamul Haq, Mohsina Haq, Ashfaq Ahmed, Hala Haq, Abbas Saleem Khan

**Affiliations:** 1 Department of Biotechnology and Genetic Engineering, Hazara University, Mansehra 21300, KP, Pakistan; 2 Institute of Biotechnology and Genetic Engineering, The University of Agriculture, Peshawar, KP Pakistan; 3 Peshawar Medical College, Riphah International University, Warsak Road Peshawar KP Pakistan; 4 Department of Microbiology, Peshawar Medical College, Riphah International University, Warsak Road Peshawar KP Pakistan; 5 Haematology, Pathology Department National University of Medical Sciences Rawalpindi Pakistan; 6 Peshawar Dental College, Riphah International University, Warsak Road Peshawar KP Pakistan

**Keywords:** Dengue, Outbreak, DENV, real-time PCR, RNA Virus, Pakistan

## Abstract

**Background:**

Dengue is a widely spread mosquito-borne infection in humans, which in recent decades declared is public health problem globally. The dengue virus contains 4 different serotypes (DENV-1, DENV-2, DENV-3, and DENV-4) which belong to the genus Flavivirus.

**Aims:**

A descriptive experimental study was conducted to determine the epidemiology, types of Dengue serotypes, clinical features, laboratory probe, and markers for primary diagnosis of dengue virus infection in hospitalized patients.

**Methodology:**

A total of 691 suspects were diagnosed from August to October 2019 in district Shangla KP, Pakistan. Serological tests were used for nonstructural protein-1 antigen (NS1), and antibodies (immunoglobulin-M (IgM) & Immunoglobulin-G (IgG)) while real-time PCR was used to confirm the cases. The data was statistically analyzed using IBM-SPSS Statistics 20 version.

**Results:**

The dengue virus infection was more prevalent in the male group (68.09%) than the female group (31.1%). A large number of patients were from rural areas (63.5%) while from urban areas were (36.4%), whereas Besham tehsil was found the most affected compared to other regions. The most prevalent serotype observed in our study was DENV-3 (56.60%) while DENV-4 was the least prevalent serotype (1.88%). Among the age-wise analysis of dengue-virus-infected individuals, the age group of 19–37 years (64.07%) was found the most affected group. The month-wise analysis revealed that the highest number of infections (49.8%) were recorded in September. Significant differences were noticed among blood parameters.

**Conclusion:**

The possible reasons for the dengue overwhelming in the study area could be less or lack of awareness particularly regarding the transmission of viral infections, improper sewage management, and no effective vector control strategies that lead the dengue outbreaks in the study population.

## Introduction

Dengue fever caused by the dengue virus (DENV) is a major public health issue and is among the most prevalent vector-borne diseases found in the tropical and subtropical regions of the world. Dengue virus is a single-stranded RNA virus of the genus Flavivirus and family Flaviviridae in Amarillovirales order transmitted by Aedes aegypti and Aedes albopictus^1^,^2^. There are four serotypes of the dengue virus from DENV-1 to DENV-4^3^. Dengue virus infection could be divided into three phases like symptoms begin with mild dengue fever (MDF) followed by dengue hemorrhagic fever (DHF) and end-with dengue shock syndrome (DSS)^4^,^5^. All the mentioned stages (MDF, DHF, and DSS), can exhibit signs of dengue fever in the clinical results of the dengue-infected person^6^. The infected individuals could represent several symptoms including high fever, headache, pain in joints, and myalgia^7^. In the last decade, Pkistan experienced huge outbreaks in which main serotypes DENV-2, DENV-3, DENV-4 were found in Punjab, nevertheless, in Swat and Shangla areas of KP province only DENV-2, DENV-3 was reported from 2013 to 2018^8^, ^9^, ^10^. Recent studies have reported that dengue virus infection is endemic in Pakistan, where the highest incidences have been reported in the monsoon season^11^, ^12^. Previously, three major outbreaks have been reported (in 2006, 2010, and 2011) with more than 40,000 individuals in the country^13^, ^14^. In 2011, a huge outbreak of dengue was experienced in Lahore in which the dual serotype infections increased the number of cases and related mortality^15^. In a study in 2017, a total of 302 dengue positive have been reported from the Mardan district^16^. Currently, the expansion of dengue virus infection from urban to rural areas and less affected regions (from most affected regions) could have been increased by 30-fold and could be experienced as emerging and re-emerging dengue-virus outbreaks^17^. The outbreak of such viral infections could be different every next year. According to the world health organization (WHO), the annual expansion of dengue infection was in a range from 0.4 to 1.3 million in the years 1996–2005^18^. In 2013, a total of 320 dengue positive cases in the Shangla and Buner districts of Khyber Pakhtunkhwa were reported^19^. Basic analytic methods, government hospitals, and private medical care centers across the country are diagnosing and administering dengue virus infection cases. However, unlike other viral infections such as polio, there is a huge gap regarding the awareness programs among the general population particularly regarding vector-borne diseases in hilly areas of the KP province^20^. The objective of the current study was to examine the serological and epidemiological aspects of dengue virus infection in the population of district Shangla and other regions of KP province, Pakistan. In the current study, we detected the dengue virus infection from serum samples using IgM capture ELISA (dengue fever virus IgM capture ELISA Focus Diagnostics, CA, USA) which is a primary screening test of dengue infection and with the real-time polymerase chain reaction (RT-PCR) during the recent outbreak.

## Methodology

### Description of study

Shangla district is located in the Malakand division, Khyber Pakhtunkhwa province of Pakistan. The district has a total area of 1,586 square kilometers. We collected samples from dengue patients admitted at Shangla DHQ Alpuri and THQ Besham, the district's two main health centers.

**Figure:**
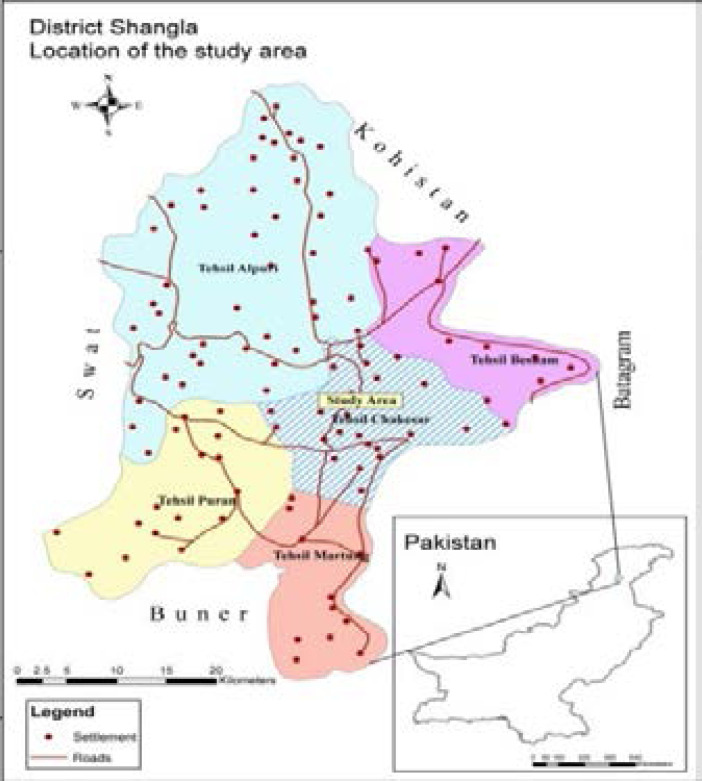
The given map represents the location of the study area District Shangla

### Data collection

The current study was conducted, and data was collected from two hospitals (Alpuri and Besham) of district Shangla during the recent epidemic of dengue virus infection. The study was conducted from August 2019 to October 2019. Ethical approval was taken from the Department of Biotechnology and Genetic Engineering at Hazara University Mansehra and the medical superintendent of District Head Quarter hospital Shangla.

### Sample size and testing criteria

A total of 691 dengue suspected individuals were examined in this study. All the patients were analyzed after the appearance of clinical signs and symptoms including fever, vomiting/nausea, body aches, skin rashes, body aches, bleeding gums, and bleeing nose. We examined various blood parameters such as platelets, white blood cells (WBC), red blood cells (WBC), hemoglobin, neutrophils, and packed cell volume (PCV). The patient with dengue signs and symptoms were confirmed by dengue virus infection confirmation tests that detect serological markers such as NS1, IgG, IgM antibodies, and RNA of dengue virus.

### Collection and Detection of NS1 Antigen

A 5ml of blood was collected from each patient in EDTA tubes and serum was obtained by sample centrifugation at 3000 rpm for 8 minutes. The serum samples were separated with early dengue NS1 detection by Enzyme-Linked Immunosorbent Assay (ELISA) (Panbio, Brisbane, Australia) as per the manufacturer's instructions^21^.

### Detection of Anti-DENV IgM and IgG Antibodies

Dengue-virus specific IgM antibodies were examined using immunoglobulin M (IgM) capture ELISA (Dengue Fever Virus IgM Capture ELISA Focus Diagnostics, CA, USA) according to the manufacturer's guideline. Dengue indirect IgG ELISA (Panbio, Queensland, Australia) was used for the detection of anti-DENV IgG^22^.

### Real-time PCR Based Viral RNA detection

Viral RNA was extracted by using 140 µL of serum sample with QIA viral Mini kit (Qiagen, Germany) as per the manufacturer's protocol. A one-step real-time TaqMan reverse transcriptase RT-PCR for detecting and typing DENVs was carried out according to methods. PCR reaction was performed for 4 serotypes i.e., DENV-1, DENV-2 DENV-3, DENV-4 following the procedure previously adopted by Johnson et al. 2005[23. The details of four serotypes specific primers and fluorophore probes are represented shown in Table 1.

**Table 1:** Serotype-specific DEN virus real-time polymerase chain reaction (RT-PCR) “Adapted from Johnson *et al* 2005, American Society for Microbiology.”

Virus Serotype	Nucleotide Sequence	bp	Fluorophore
DENV-1 F	CAAAAGGAAGTCGTGCAATA	20	
DENV-1 R	CTGAGTGAATTCTCTCTACTGAACC	25	
DENV-1 Probe	CATGTGGTTGGGAGCACGC	19	FAM/BHQ-1

DENV-2 F	CAGGTTATGGCACTGTCACGAT	22	
DENV-2 R	CCATCTGCAGCAACACCATCTC	22	
DENV-2 Probe	CTCTCCGAGAACAGGCCTCGACTTCAA	27	HEX/BHQ-1

DENV-3 F	GGACTGGACACACGCACTCA	20	
DENV-3 R	CATGTCTCTACCTTCTCGACTTGTCT	26	
DENV-3 Probe	ACCTGGATGTCGGCTGAAGGAGCTTG	26	TR/BHQ-2
DENV-4	TTGTCCTAATGATGCTGGTCG	21	
F			
DENV-4 R	TCCACCTGAGACTCCTTCCA	20	
DENV-4 Probe	TTCCTACTCCTACGCATCGCATTCCG	26	Cy5/BHQ-3

### Statistical Analysis

Statistical analysis was done using IBM-SPSS version 20 to examine the data. The study sample was described using monovariate analysis. The difference between mean peripheral blood parameters in the acute and critical phases was investigated using the Student's t-test.

## Results

Using various assays for detection of dengue virus A total of 691 dengue suspected patients' blood was tested by different detection techniques 373 were confirmed dengue positive (53.97%). The suspects were examined with NS1, IgM, and IgG procedures confirmed (71.5%) dengue-positive cases. In which 113 (45.1%) subjects were positive for NS1, while 81 (47.3%) were detected positive for IgG, and 73 individuals were detected positive for anti-dengue IgM antibodies. For further confirmation, 267 dengue positive samples are analyzed with RT-PCR, of which 106 out 267 were confirmed positive(39.70%) as shown in Table 2.

**Table 2:** Positive cases detection by various techniques

Assay	Positive cases	Percentage
NS1	113	45.1%
IgM	73	46.7%
IgG	81	47.3%
RT-PCR	106	39.70%
Total	373	N/A

### Distribution of Dengue serotypes prevalence for RT-PCR

A total of 106 cases, which were detected positive using RT-PCR, out of which, DENV-1 serotype was (4.71%), DENV-2 (36.79%), DENV-3 (56.60%), and DENV-4 was (1.88%) (given in Table 3).

**Table 3:** Distribution of dengue serotypes confirmed by RT-PCR

Serotypes	Total positive	Percentage (%)
DENV-1	05	4.71
DENV-2	39	36.79
DENV-3	60	56.60
DENV-4	02	1.88
Total	106	N/A

### Tehsil wise distribution of dengue fever infection in district Shangla

**Figure 1:**
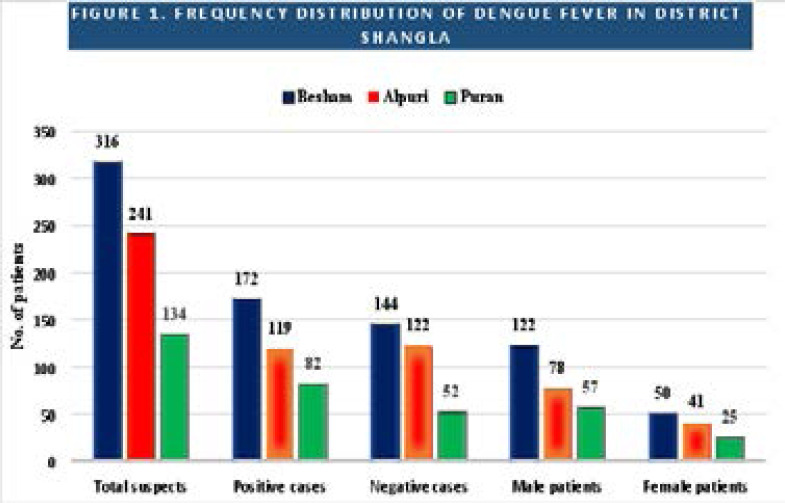
Dengue fever distribution among three tehsils (Alpuri, Besham, and Puran) of the Shangla district is represented. The blue color represents the frequency of dengue virus infection in Besham while the red and green represent the frequency of cases in Alpuri and Puran, respectively.

A total of 691 dengue suspects were studied from different tehsils of the shangla district, including cases from Besham 316, Alpuri 241, and Puran 134. Furthermore, high severity was noted in Besham 172 (54.43%), followed by Alpuri 119 (49.37%) and Puran 82 (61.19%) as shown in Figure 1).

### Area-wise distribution of dengue severity

In the area-wise severity, more patients were from rural areas 237 (63.5%) as compared to the urban areas 136 (36.4%) as represented in Table 4.

**Table 4:** Demographic representation of dengue virus infection

Suspected cases	Confirmed cases	Negative cases	Male patients	Female patients
691	373 (53.9%)	318 (46.1%)	257 (68.9%)	116 (31.1%)
Areas	Rural areas	237 (63.5%)	Urban areas	136 (36.4%)

### Dengue infection prevalence among different age groups

We examined the age-wise prevalence of dengue virus infection. The patients were classified into the following age groups; 1–18 years, 19– 37 years, and 38–60 years. The middle age group (19–37 years) was observed to be the most affected age group with the highest prevalence (64.07%) followed by age group 1–18 years (25.47%) and 38–60 years (10.45%) as given in Table 5).

**Table 5:** Dengue outbreak among different age groups

Age-group	Suspects	Confirmed cases	Percentage
1–18 years	164	95	25.47%
19–37 years	374	239	64.07%
38–60 years	149	39	10.45%
Total	691	373	--

### Clinical presentation

In the sight of clinical manifestation, all the patients were identified with mild to severe fever (100%), where body aches were detected in 93.1% of the patients. About 77.47% of patients exhibited the symptoms of vomiting/nausea while 43.96% exhibited enlarged liver. Some other complications such as skin rashes (26%) and bleeding (5.63%) were also observed as shown in Table 6).

**Table 6:** Clinical manifestation of dengue-severity observed in positive patients

Fever	373	100%
Vomiting/nausea	289	77.47%
Body aches	347	93.1%
Skin rashes	97	26%
Nose and/or gum bleeding	21	5.63%
Enlarged liver	164	43.96%

### Monthly distribution of dengue fever

**Figure 2:**
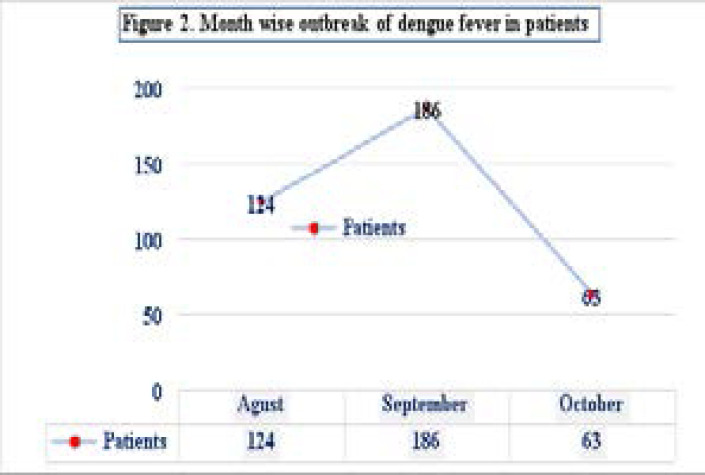
Month-wise representation of dengue cases

The frequency of positive cases in August, September, and October was examined. The highest prevalence was observed in September with 186 cases (49.8%) followed by August 124 (33.2%) and October 63 (16.8%) as represented in Figure 2.

### Analysis of blood parameters

In our study, a total of 691 subjects were categorized primarily into Dengue Fever (DF) and Dengue Hemorrhagic Fever (DHF). Other conditions that might induce complete blood count alterations, such as hematological and reticular endothelial problems, as well as individuals using cytotoxic medications and Patients who experienced dengue-related complications such as acute liver failure or myocarditis were excluded from the study. All of the patients were treated according to Sri Lankan government standards for the treatment of DF and DHF. Both DF and DHF could lead to alteration in blood parameters. All data of peripheral blood parameters during the day 1st to 3^rd^ day (acute and febrile phase) and 5^th^ day (critical phase) among leakers and non-leakers respectively were investigated from the blood specimen of the patients. In the acute phase (day 2, and 3) of the infection, probability values were predicted which are as shown in Table 7.

**Table 7:** Distribution of FBC throughout the acute febrile phase (day 2 to 3 of sickness)

Blood parameters	Fever stages	Mean	Std- Deviation	P-value	Ranges for blood parameters;
Platelets	DF= 181	69734.8	22426.8	p = 0.04*	150–400*103/µL
	DHF = 173	27624.2	22956.5		
WBCs	DF = 181	7.1	3.70	P = 0.68	4.3–10*103 /µL
	DHF =173	3.8	3.73		
Neutrophil	DF = 181	2.8	1.03	P = 0.61	1.8–7.0*103/µL
	DHF =173	1.2	0.53		
Lymphocyte	DF = 181	1.9	0.43	P = 0.05	1.0–4.8*103/µL
	DHF =173	0.9	0.43		
Hemoglobin	DF = 181	14.4	2.27	P = 0.08	13–18*106 /µL
	DHF =173	17.0	2.35		
PCV%	DF = 181	39.4	4.40	P = 0.05	38–50%
	DHF =173	39.0	4.00		

* Significant value

Leucopenia predicted a p-value of 0.68 for DF patients and DHF patients where the p-value for neutrophils was 0.61. A significant association of Hemoglobin and mean packed cell volume (PCV) with the disease conditions was observed with the p-value=0.08 and p-value=0.05, respectively. Thrombocytopenia (platelet<69,000 cells/mm^3^) was observed in 51.23% dengue infected patients. Similarly, the blood samples of the 5^th^ day of the illness were considered among the leakers and non-leakers, for the peripheral blood parameters in the dengue patients. Leucopenia in the critical phase was associated with the stage of disease (p-value=0.04) as represented in Table 8.

**Table 8:** Distribution of FBC on (day 5) of illness considered among leakers and non-leakers

Blood parameters	Fever stages	Mean	Std- Deviation	P-value	Ranges for blood parameters
Platelets	DF = 44	67454.5	22501.1	P = 0.02 *	150–400*103 /µL
	DHF = 130	25500	21184.0		
WBCs	DF = 44	4.4	2.23	P = 0.04*	4.3–10*103 /µL
	DHF = 130	2.6	2.07		
Neutrophil	DF = 44	2.8	0.78	P = 0.51	1.8–7.0*103 /µL
	DHF = 130	1.4	0.53		
Lymphocyte	DF = 44	1.9	0.44	P = 0.3	1.0–4.8*103 /µL
	DHF = 130	1.0	0.37		
Hemoglobin	DF = 44	15.6	2.44	P = 0.01*	13–18*106 /µL
	DHF = 130	14.4	3.20		
PCV%	DF = 44	42.3	2.96	P = 0.1	38–50%
	DHF = 130	38.2	2.87		

There was no significant association of neutrophils and lymphocytes with the stage of disease, however, variation Hemoglobin and mean packed cell volume (PCV) were highly associated with the stage of disease (p-value= 0.01). Thrombocytopenia (platelet<69,000 cells/mm^3^) was associated (p-value= 0.02) with the critical phase of the infection.

## Discussion

Dengue virus infection is among the top emerging and re-emerging viral infections leading to millions of deaths every year throughout the world^24^. It is believed that the dengue virus could have come to Pakistan via contaminated eggs of dengue vector on tires in the seaport of Karachi^25^. The first cases of dengue infection in Pakistan were documented in Karachi Sindh Pakistan in 1994. A total of 15 patients were examined who represented positive anti-dengue antibody (IgM) and found with DENV-1 as well as DENV-2 infection^26^. The burden of dengue virus infection was then spread to the Baluchistan province of Pakistan in the year 1995^27^. Since then, the infection was reported with all serotypes (DENV-1 to DENV-4) from the entire country. In the current study, we examined 373 (53.9%) subjects and observed that all the subjects were infected by the dengue virus. Among the total examined subjects, a prevalence of 68.9% was reported in the male individuals which is more than the female patients (31.1%) as represented in Table 4 and Figure 1. The gender-wise prevalence in our study is following the previous studies^28^. We have reported that most of the cases (64.07%) were from the age group of 19–37 years, followed by (25.47%) patients and 1–18 years as represented in Table 5. The higher prevalence in the middle age group could be attributed to the higher exposure of these individuals to the environment leading to an increase in the chances of encountering the vector. Further, traveling exposure for business and other purposes could also increase the risk of infection. The other age groups which are mostly children and older individuals have very less exposure to the environment comparatively. A previous study has also reported the middle-aged population is the most affected population^29^. Further, the patients were documented with the symptoms of dengue infection such as fever (100%), inner bleeding, and enlarged liver 43.96% as mentioned in Table 6. According to a previous study, liver wounds in dengue disease could be due to the direct influence of the virus or host immunity with liver cells, which could become a facet for vascular leakage inside the liver^30^.

In the current study, the patients were initially screened for the presence of dengue antigen such as NS1 which was a lower detection (45.1% sensitivity) compared to anti-dengue antibodies such as IgM with (46.7%) and IgG with (47.3%), while, RT-PCR has higher sensitivity (39.70%). In our study, more patients were identified with the serotype (DENV-3) compared to other serotypes. However, we have also observed the serotype DENV-4 identified in some patients for the first time in the study population, which might be due to migrants (dengue-infected) traveling from the dengue-endemic areas. According to WHO, the clinical presentation that ensures the sign and symptoms could serve as an integral method for identifying the dengue virus infection^31^,^32^. With the area-wise severity, more patients were documented from the rural areas 237 (63.5%) than the urban areas 136 (36.4%). Previous studies have also reported similar findings regarding the occurrence of infections in different areas^33^,^34^. According to a report, dengue escalation from urban to rural areas and its expansion to new regions reported a 30-fold increase in the occurrence of the outbreaks^35^. Likewise, the monthwise report as represented in Figure 1 represents September with the largest number of cases (49.8%) followed by August (33.2%) and October (16.8%). This could be explained by the fact that the post-monsoon season could be the most suitable season for mosquito proliferation hence increase the risk of vector-borne infections. Additionally, the District Shangla is mostly comprised of rural areas. The stagnant water and muddy puddles in the region could also be a major reason for the proliferation of dengue mosquitoes, and their expulsion during the rainy days. As reported previously, the dengue virus infection rate has been high in the post-monsoon season compared to the monsoon season^36^.

The total Blood parameters counted as thrombocytes were noted lower in patients with the secondary dengue infection where a decrease in the number of thrombocytes was observed in patients detected positive for IgG. A significant difference was observed between different stages of the disease and variation in blood parameters. Previous investigation has reported that fever stages such as DF and DHF could be identified by platelets examination^37^. There was no significant association in the WBCs, neutrophile, lymphocyte, hemoglobin, and packed cells volume percentage (PCV %) as we observed a p-value > 0.05 (given in Table 7). There were no significant differences between the mean values of the peripheral blood parameter, however, in some cases, the increased values were also observed38. In other words, the increase was observed among leakers and non-leakers screened on day 5^th^ of the illness. A significant association of thrombocytes (p-value=0.02) with DHF and DF was observed. Hemoglobin of DF and DHF patients with (p-values> 0.01) represented in Table 8 indicated a significant association with the phase of infection at 4th and 5th days. A similar study described that the DHF could be categorized by examining the density of hemoglobin^39^. While Leukopenia count in DF and DHF patients was observed with their means less than 500 cells/mm^3^. Furthermore, WBCs and neutrophils values in the acute phase of dengue infection were observed with no statistical difference in DHF patients^40^.

## Conclusion

In this study, a total of 373 dengue positive cases were identified with NS1 identification assays initially and were confirmed with the highest sensitivity (93.8%) RT-PCR. DENV-3 was the most common serotype (56.60%), followed by DENV-2 (35.84%), DENV-1 (4.71%), and DENV-4 was (2.83%) in our study. We have reported the serotype DENV-4 for the first time in the study population. Besham tehsil was the most severely affected (n=172) among others. Male individuals were more affected (68.9%) than females (31.1%), and the severity was most common among the age group range 19–37 years as (64.07%). The most common symptom was fever (100%) where the infection rate was higher in September (49.8%). A significant difference in blood parameters was noted at the 4th and 5th (critical phase) days of infection compared to the initial days. The current study shows that an incidence of a dengue epidemic could further increase the risk of DENV-4 prevalence as observed in some patients. Because of the remote location of the study area and lack of education, we suggest that increased screening tests, public awareness regarding the potential risk factors, and prevention measures for dengue infection are urgently needed.

## Data Availability

The authors confirm that the data supporting the findings of this study are available within the article.
